# Hands-on approach during breastfeeding support in a neonatal intensive care unit: a qualitative study of Swedish mothers' experiences

**DOI:** 10.1186/1746-4358-1-20

**Published:** 2006-10-26

**Authors:** Lena Weimers, Kristin Svensson, Louise Dumas, Lars Navér, Vivian Wahlberg

**Affiliations:** 1 Department of Pediatrics, Karolinska University Hospital Huddinge, Stockholm, Sweden; 2 Department of Obstetrics, Karolinska University Hospital Solna, Stockholm, Sweden; 3 Department of Women and Child Health, Division of Reproductive and Perinatal Health Care, Karolinska Institutet, Stockholm, Sweden; 4 Department of Nursing Sciences, Université du Québec en Outaouais, Québec, Canada; 5 Division of Pediatrics, Department of Clinical Sciences, Intervention and Technology (CLINTEC), Karolinska Institutet, Stockholm, Sweden; 6 Ersta Sköndal University College Department of Health Care Sciences, Stockholm, Sweden

## Abstract

**Background:**

Assisting mothers to breastfeed is not easy when babies experience difficulties. In a neonatal intensive care unit (NICU), nurses often help mothers by using hands-on-breast without their permission. Little is known about how mothers feel about this unusual body touching. To gain more knowledge from mothers who lived through this experience, this hands-on practice was studied in a NICU in Sweden.

**Methods:**

Between January and June 2001, in-depth interviews were conducted with ten mothers of preterm or sick term infants and all of them experienced the hands-on approach. In this research, Radnitzky's seven principles of hermeneutic interpretation were applied in order to interpret the meaning of mothers' responses. This article presents results related to the period of initiation of breastfeeding. This qualitative study was based on a combination of the models of Gustafsson, Orem, and Aarts' Marte Meo.

**Results:**

Five main themes were identified: Insult to integrity, Manipulating the baby, Understanding and adjustment, Breasts as objects, Alternatives to this practice. Hands-on help in the breastfeeding situation was experienced as unpleasant and the women experienced their breasts as objectified. The mothers accepted the hands-on help given by nursing staff, even though they considered it unpleasant. Most mothers expressed a need for assistance when starting breastfeeding, but could not suggest any alternative to hands-on help such as demonstrating with an artificial breast and a doll.

**Conclusion:**

The study provides information about how mothers experience unexpected hands-on help with breastfeeding in a NICU, which has not been described previously. Since most mothers in this study regarded this behavior as unpleasant and not helpful mostly because it was unexpected and unexplained, it would be important to either explain beforehand to mothers what type of physical approach could be attempted on their body or better, to avoid this type of approach completely.

## Background

Mothers of preterm or sick infants face specific difficulties related to early mother and baby separation in a time of crisis and disillusion; this leads to a lack of appropriate bonding experiences [1,2]. The infants being sick or immature, the initiation of breastfeeding often means repeated failures [2]. Failures in breastfeeding can lead to anxiety, low self-confidence, and low self-esteem [3,4], and feelings of being a bad mother [5]. In these instances, the nursing staff's knowledge, attitudes and skills have a great impact on the mothers' self-confidence and ability to initiate breastfeeding [6,7]. Self-confidence has been shown as an important predictor for breastfeeding duration [8].

Midwives and neonatal nurses who lack knowledge in breastfeeding management experience difficulties in giving timely and adequate information, and in supporting mothers [9]. In fact, breastfeeding frequency is increased when nursing staff have theoretical and practical knowledge of good breastfeeding technique [6,10,11]. Hedberg-Nyqvist and her colleagues also demonstrated that uniformity of language and approach from nursing staff increase the professional breastfeeding support to mothers [12]. Duffy and colleagues have illustrated that professional help with positioning and latching-on to the breast is of importance not only prenatally but also soon after birth, for mothers to understand the "mechanics" of breastfeeding and to readjust immediately incorrect, painful, or ineffective positioning [11]. Different approaches have been used to support mothers during this learning experience such as verbal or visual descriptions [12-14] and demonstrating with an artificial breast and doll [11,13,14,16,17]. Our clinical experience also tell us that some nurses use a hands-on approach on their own body or on the mother's breast.

Two recent studies in UK and Australia have described negative effects of hands-on assistance by staff in breastfeeding situations [13,14]. They showed that the mothers who were visually instructed in breastfeeding technique, with an artificial breast and a doll, experienced fewer breastfeeding problems, were more likely to breastfeed, and even breastfed longer than those who were subjects of the hands-on approach. Nothing is known about how Swedish mothers experience this type of approach. As Sweden is recognized as a leading breastfeeding culture in a developed country, it is of interest to know if Swedish mothers react similarly to this touching of the breast by the staff. It is of special interest also to study this phenomenon with mothers of babies with expected breastfeeding problems such as sick or preterm infants in a NICU.

This article reports on the mothers' experiences with hands-on support by the nurses when their baby is in a NICU. It originates from a larger study in which informative and supportive nursing strategies were examined as potential factors to increase mothers' self-care capacities [18].

In Sweden, most mothers-to-be participate in prenatal education groups where they normally learn about breastfeeding basics and day-to-day management. In fact, Swedish prenatal teaching content respects international recommendations such as those from the Baby Friendly Hospital Initiative (BFHI) [19,20]. As in other countries, the quality of information depends on midwives' attitudes, knowledge, and skills. Living in a country where the vast majority of women initiate breastfeeding, they also can benefit from the practical knowledge of mother-to-mother breastfeeding groups (Amningshjälpen). Almost all parents' magazines present a positive view of breastfeeding and advertisements usually respect the International Code of Marketing of Breast Milk Substitutes [21]. Generally speaking, breastfeeding presents no problem to most Swedish mothers since they should be knowledgeable about the basics of breastfeeding from antenatal groups, magazines, and breastfeeding groups.

Mothers of sick babies or preterm infants are the exception. Very often, either they did not participate in breastfeeding classes since they gave birth too early, or what they have learned doesn't apply to their particular situation, their babies presenting complications with the initiation of breastfeeding. Infants in a NICU also often have been fed in many different ways before the actual initiation of breastfeeding, such as tube, cup, or bottle feeding. We know that breastfeeding frequency is enhanced when the baby first sucks from the breast rather than from a bottle [22]. We also recognize the importance of skin-to-skin contact for both mother and baby for the initiation of breastfeeding [23-26], and especially when the infant is premature [1,27,28]. All these have to be taken into consideration when sick or premature babies are to initiate breastfeeding. Their mothers need not only good explanations and adequate guidance, but also specialized support to help them with breastfeeding [29,30]. This professional service should also be in line with the same BFHI recommendations pertaining to mothers' teaching, but obviously need to be adapted to the condition and maturity of the infant. Weimers and Nyström have produced an adapted guide for the Ten Steps to Successful Breastfeeding in the NICU [31]. Other studies have demonstrated that breastfeeding rates can be increased when applying the Baby Friendly Initiative program in the NICU [32-34].

The importance of maternal confidence and knowledge in the success of breastfeeding is well recognized [15,16,18]. What is not so well understood is how this self-confidence can be enhanced or hindered during the first breastfeeding experiences. We believe that some approaches, such as hands-on support by the nurse, could be negative to the development of a mother's self-confidence when initiating breastfeeding, and especially in the NICU where this could be particularly difficult if the baby is either sick or premature.

**Figure 1:**
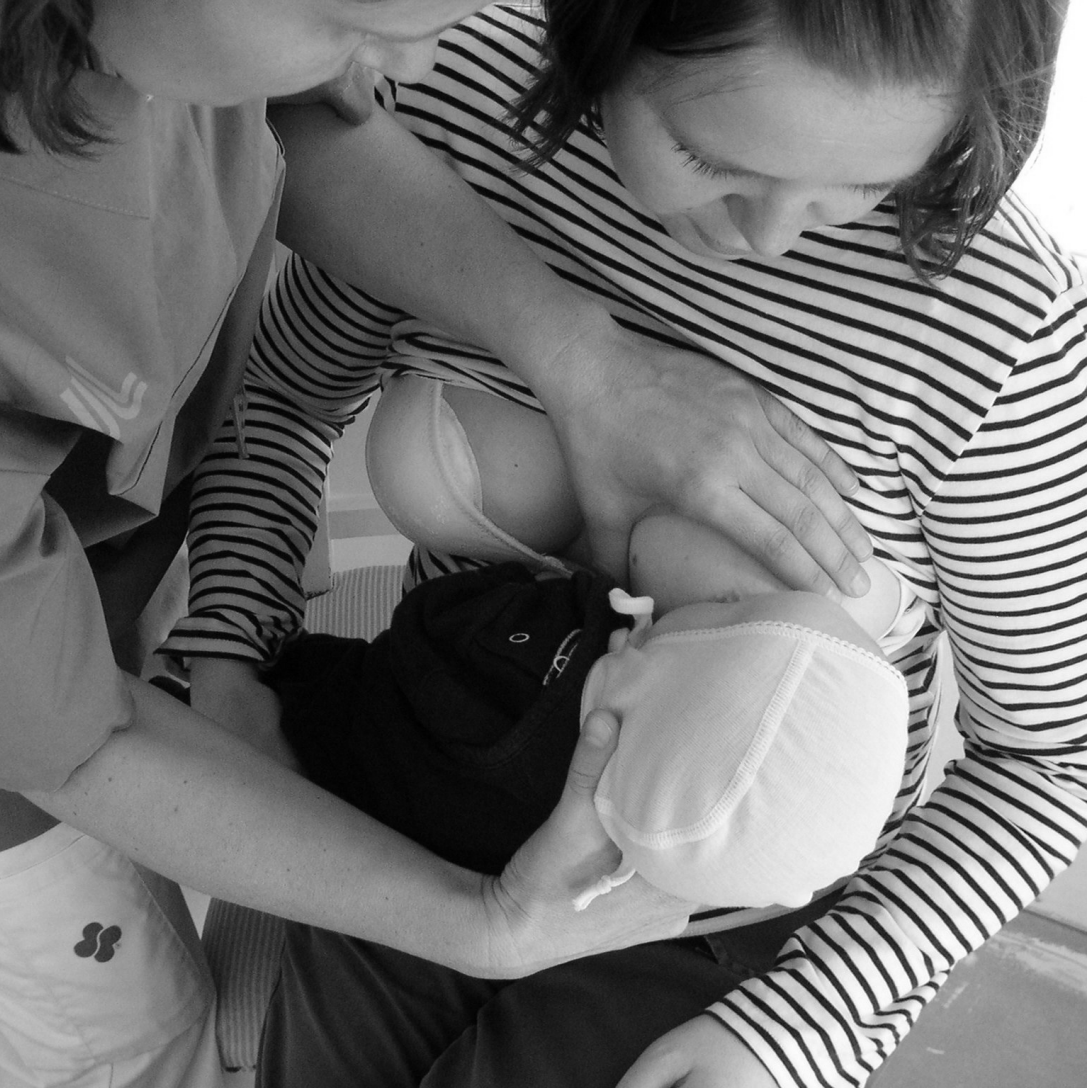
A teaching situation: Nurse giving hands-on help.

"Hands-on-breast" assistance by the nurse is even more common when sick and preterm babies initiate breastfeeding; unexpectedly, that is, without asking permission of the mother, nurses grasp the mother's breast with their own hands to place it into the infant's mouth, and they also help the mother with manually milking the breast for milk expression. In this study, hands-on help is what is meant when nursing staff unexpectedly and actively touch the mother's breast and/or the baby's head, neck, or back with their hands to get the baby to the breast or when the nurse touches the breast during manual milking of the breast (See Figure 1). As explained by Ingram and colleagues from the United Kingdom [13] and Fletcher and Harris from Australia [14], we believe that a mother would benefit more from a "hands-off" approach to reinforce her self-confidence since she would learn to put the baby to the breast herself rather than having the infant latched-on for her. However, this is only presumed to be similar for Swedish mothers since no study is reported on this subject. One of the objectives of this study was to reveal Swedish mothers' experiences of hands-on nursing approach to support breastfeeding initiation in a NICU.

## Methods

### Research question

How do mothers experience support by nursing staff in breastfeeding situations while their baby is in the NICU? This was the general question underlying this qualitative study. We report here only the sub-question pertaining to the hands-on approach: How do mothers experience hands-on support by nursing staff in breastfeeding situations while their baby is in a NICU?

### Sample

Between January and June 2001 two independent nurses from the NICU at the selected hospital alternately informed potential mothers about the study. To be included in the study, mothers had to understand Swedish but not necessarily be of Swedish origin, to have given birth to either a premature infant or a sick baby requiring to be hospitalized in the NICU, and to be breastfeeding. Also, according to the inclusion criteria, 50% of the babies should be full term sick infants and 50% premature, in order to represent the two main patient categories in the NICU. There was no preset number of mothers; the researchers included any interested mother until saturation of data was reached.

### Procedure

The mothers were recruited in a NICU at a university hospital in Stockholm, Sweden. This NICU consists of four rooms accommodating a total of twenty babies, two isolation rooms where parents can stay, and two rooms exclusively for parents. As for background data per representativeness of the sample, during 2001, 564 newborns had been admitted to this unit, 50% were premature babies. The staff consists of nurses, pediatric nurses, and nurse-midwives who have responsibility for these babies and their parents, including professional assistance with breastfeeding. Pediatricians and one nutritionist are also important professionals in the team to assist with nutrition aspects.

Qualitative in-depth interviews were used to elicit mothers' experiences with breastfeeding support. The interviews were conducted after discharge of the baby, either at home or elsewhere, as per the mother's choice. The interviews were conducted by the researcher according to a pre-determined semi-structured guide (see Table 1); they were audio-recorded and field notes were documented. All information was confidentially handled as per the rules of the Karolinska Institutet Research Ethics Committee which approved this study (Dnr 460/00). Each interview lasted between 30 and 90 minutes and all were performed within a six month period. An interview outline was used, based on the chosen theoretical frameworks which guided this study, but this was only a guideline since we were interested in the inductive method of describing mothers' experiences and not their answers to specific closed questions.

**Table 1:** Semi-structured interview guide

1. Can you tell me about your breastfeeding experiences in NICU during the first days after birth? Tell me about your experience of the support you received from the nurses during your initial breastfeeding situations?
2. Did the assistance, the information, and the support you received in NICU, influence your attitude towards breastfeeding? Did it influence your breastfeeding experience for you and your baby? Do you think it matters how nurses help you and your baby in a breastfeeding situation?

3. Did you receive hands-on assistance from the nurses in NICU when you were attempting to breastfeed? If yes, how did the baby react? How did you feel about this at that time? At this moment thinking back, have your feelings altered towards hands-on assistance?

4. Did you experience that you needed hands-on assistance from the nurse in breastfeeding situations? Would an artificial breast and a doll have been an alternative when guiding you instead of the hands-on approach?

5. Can you describe what an ideal teaching or support breastfeeding situation would be like for you in NICU? What would be important for you? What should the nurses take into consideration when guiding you?

As supplementary addition to each question 1–5; Can you describe in more words what you mean? Do you have any comments, questions about the subjects we have just discussed? Have I understood you correctly?

### Theoretical framework

The study was based on three intermeshed theories: Gustafsson's SAUK model [35,36], Orem's self-care theory [37,38], and Aarts' Marte Meo pedagogical approach [39]. In the breastfeeding support interventions, these three theories complement each other.

Gustafsson describes the interrelations which should take place between a professional and a client, influencing self-knowledge and self-esteem of both parents and staff. This "confirming care" is of importance especially when we are vulnerable, so here it is particularly relevant for parents who are very emotional with their premature or sick newborn. The abbreviation (SAUC) for this model stands for Sympathy-expressing, Acceptance-establishing, Understanding, Competence-manifesting [35].

Very much linked to the Gustafsson's model is the self-care model from Orem [37]. In her description of the nurse's role, Orem insists on actions empowering parents who act as self-care agents for their dependent newborn. Nurses can help parents understand the baby's experiences, so they can gradually learn how to take responsibility for this new baby, with actions to encourage their self-knowledge and their self-confidence as new parents.

In supporting positive actions from the parents, nurses can reinforce their self-confidence in caring for this fragile baby; this is the basis of the Aarts' pedagogical model. In fact, according to Aarts, the Marte-Meo method should be used as a code of conduct with the parents [39]. For her, the very first step consists of increasing parents' knowledge so to help them feel competent and essential to their baby's health from this very moment.

### Data analysis

Interviews were transcribed from their audio-recorded form onto paper using Word software. Then, Radnitzky's principles for hermeneutic interpretation were used to analyze those qualitative data [40]. For this type of analysis, the person analyzing data should have knowledge about the subject being analyzed and make sure she is open to neutrality of the data as well as to interpretation involving constant renewal and creativity. The interpretation is considered complete only when the text is well integrated and does not contain any logical contradictions. This model extends understanding from what is called a hermeneutic circle or coil. The interviews are carefully analyzed to identify significant clusters of information, sequences of meaning under the form of themes and sub-themes. Theme interpretations are given to these clusters of information and then tested against the text as a whole or against other texts. The text should be understandable from its own context, not needing any additions from other sources or contexts. Mothers should be included into the study until saturation of data is reached, i.e. until there is repetition of what has already been revealed by the mothers in the study.

In this present research on the experiences of mothers with support given by the NICU staff during breastfeeding situations with their premature or sick baby, fifty initial themes were identified, later on reduced to ten main themes after discussions with experts and close comparison of data from the interviews. This article only focuses on one of these main themes: the hands-on approach of the staff in supporting mothers to breastfeed. From the analysis of data and following lengthy discussions with experts, this main theme was revealed from the interviews into five sub-themes: Insult to integrity, Manipulating the baby, Understanding and adjustment, Breasts as objects, Alternatives to hands-on support.

### Credibility of the findings

Lincoln and Guba refer to credibility as the confidence one can have in the neutrality and conformity of the results to the message of the informants [41]. Tobin and Begley insist on the importance of establishing credibility and confirmability [42]. So, in order to avoid biases during the analysis, an expert researcher has listened to tapes, read a transcription of the interviews, and compared them; the reviewer agreed that the written texts corresponded to the recorded interviews. Afterwards, initial identification of themes and sub-themes was done by the principal investigator. Two other researchers agreed on the results of the analysis of a random selection of 50 % of the written interviews. Quotes from the mothers themselves are presented as examples of the results and they serve to illustrate the discussion.

## Results

Sixteen mothers were informed about the study and twelve agreed to participate. The four mothers who refused to participate did not give reasons for their refusal. Twelve mothers answered all questions from the semi-structured questionnaire, including two mothers who did not experience the hands-on approach. We did not wait for the 50% of mothers from each category as intended since we had already reached saturation of data with 12 mothers. This article deals only with the ten mothers who have experienced hands-on approach. Eight of the ten mothers were of Swedish origin and two were of other ethnic background. The mothers were aged 26 to 35 years; five were multiparas and five primiparas. Seven mothers had given birth to premature newborns including three mothers who had twins. Three mothers of sick term infants were also recruited in order to have representation of infants from each category of babies in the NICU. The mother-child pairs are briefly described in table 2. At the time of the interviews, eight mothers were breastfeeding, three of them exclusively. Two were not breastfeeding.

**Table 2:** Description of the sample of "mother-child dyads"

**Age of mother**	**Previous children**	**Preterm infants**	**Twins**	**Full term infants**	**NICU Stay**	**Hands-on help**	**Age of infants at interview**	**Breastfeeding at interview**
26 y.o.	No			X	2 weeks	Yes	2 1/2 month	Exclusive breastfeeding
29 y.o.	No			X	2 days	Yes	2 month	Exclusive breastfeeding
32 y.o.	Yes	X	X		1 month	Yes	3 month	No
35 y.o.	Yes	X			2 weeks	Yes	2 month	Partial breastfeeding
29 y.o.	No	X	X		2 month	Yes	3 month	Partial breastfeeding
35 y.o	Yes	X			1 month	Yes	2 1/2 month	Exclusive breastfeeding
32 y.o	Yes	X			2 month	Yes	4 month	Partial breastfeeding
31 y.o	No	X	X		4 month	Yes	4 1/2 month	Partial breastfeeding
34 y.o	Yes	X			1 1/2 month	Yes	4 month	Partial breastfeeding
29 y.o	No			X	2 weeks	Yes	3 month	No

Note: Sick full term infants who are admitted to the NICU usually suffer from hypoglycemia, infection or are under observation after a complicated birth.

Results have been subdivided into sub-themes: Insult to integrity, Manipulating the baby, Understanding and adjustment, Breasts as objects, Alternatives to hands-on approach.

### Insult to integrity

Mothers felt they needed support and assistance when starting to breastfeed their premature or sick newborn, especially on how to hold the baby and to offer the breast so the baby could latch-on as easily as possible. Eight out of ten mothers described how annoyed they felt when nurses gave hands-on help without information in advance.

"... to be honest, she went straightforward and took my breast, and pushed it into the baby's mouth. You are nice and do not say anything even though you are boiling from anger when she does that. She does not ask and I do not want her help. You say perhaps mildly that it is okay, I could do this by myself ..."

These mothers said they experienced the hands-on assistance as slightly brutal, unpleasant, and that it violated their integrity. Two mothers explained that older nurses, with much clinical experience, often helped using the hands-on approach. Mothers said it mattered more to them if they trusted the person who was helping.

"... she tried to open the mouth ... it was okay that some did this but it was difficult for me when I did not know the staff well ... I think they should have asked ... they pushed the head until he, from my point of view, was almost suffocated ... it did not help at all."

Two mothers did not regard the hands-on help as unpleasant. They considered it valuable and thought it was easier to know how to do it if you had seen someone else doing it.

"...I think it was quite good to get some help. Perhaps it would have been nice if we had talked about it before."

Four mothers reported the importance of how nurses supported them but how they sometimes felt that their intimate territory was not respected.

"... it was as if she (the nurse) went over a line. She sat too near to me and disturbed me when I practiced (breastfeeding). She stood too long and inspected in some way."

Mothers gave suggestions on how to improve breastfeeding support. All of them said it was important that the staff asked before they gave the hands-on help.

"... it might have been better if they had spent more time and had sat down. He (the baby) could have the opportunity to find the breast by himself and I could have seen how he works, if he needed help and in what way, instead of pushing him there at once."

### Manipulating the baby

Mothers who had received hands-on help spoke about when their babies were learning to suck. One experience that seems to have been negative to eight mothers was when the nurse squeezed the breast into the baby's mouth. The mothers reported this as a strange feeling, and they did not want it to be repeated.

"... the first times ... I wondered what they were doing ... they just pushed the breast into her mouth. It surprised me that they did this at all... It was mostly in the beginning it was slightly unpleasant ... it was a strange feeling."

Three mothers reported that the nurse pushed one of the child's arms behind the back while they were breastfeeding. The mothers did not understand the reason for this, as they thought the baby liked to have the arm at the breast while sucking. Seven mothers also felt exposed when other persons and staff were around while the nurse was touching them with her hands on their breast.

"... you are so exposed in some way. I thought this was bothersome ... I thought it felt too private when people were around, other mothers and fathers, and they pulled and pushed the breasts any way."

### Understanding and adjustment

Eight mothers said they felt shy from the start, when expected to breastfeed in the nursery in front of parents and nursing staff. They described how they got used to this after a while. In the NICU, mothers were breastfeeding in a room shared by five babies and their parents as well as the nursing staff. Mothers report that they understand nurses doing this hands-on in front of everyone since they were used to it and they felt it was normal.

"... it might be their way of doing it, to get you going with the breastfeeding ... but, I do not like it, It might be difficult to do it in another way. I do not think it is funny when someone is pulling your breast, but you have to accept the situation."

Most of the women said they did not understand why the physical help was given. They really wondered about its purpose. They stressed that as time went by, they grew dependent on staff for assistance in breastfeeding, and sometimes wondered how they would manage when the staff would be no longer available.

The two mothers who did not dislike the hands-on approach explained this type of help suited them but they could understand that other mothers would not appreciate it.

"I do not care if someone touches me. If I think of other mothers I met there, who were not so self-confident, they might have thought it more pleasant to look first and then try."

### Breasts as objects

Five women felt that their breasts did not belong to them, that they were not parts of their body, more like inanimate objects. Two mothers even reported that they developed a new relationship with their breasts when they started to breastfeed. When the nursing staff touched the breasts, two mothers felt that their breasts were transformed into objects rather than a part of themselves.

"... my breasts were changed ... they felt more as objects. It was somewhat unpleasant but it felt strange to touch them by yourself afterwards ... I have still not recovered the feeling that everything is normal ..."

### Alternatives to hands-on support

All mothers expressed a need for assistance when starting breastfeeding, but were not positive about the use of hands-on approach. The mothers did not recognize that alternatives to hands-on help in the breastfeeding situation existed.

"It was good that they showed it but the situation is not so funny because you are so exposed, but I do not think there is any alternative."

**Figure 2:**
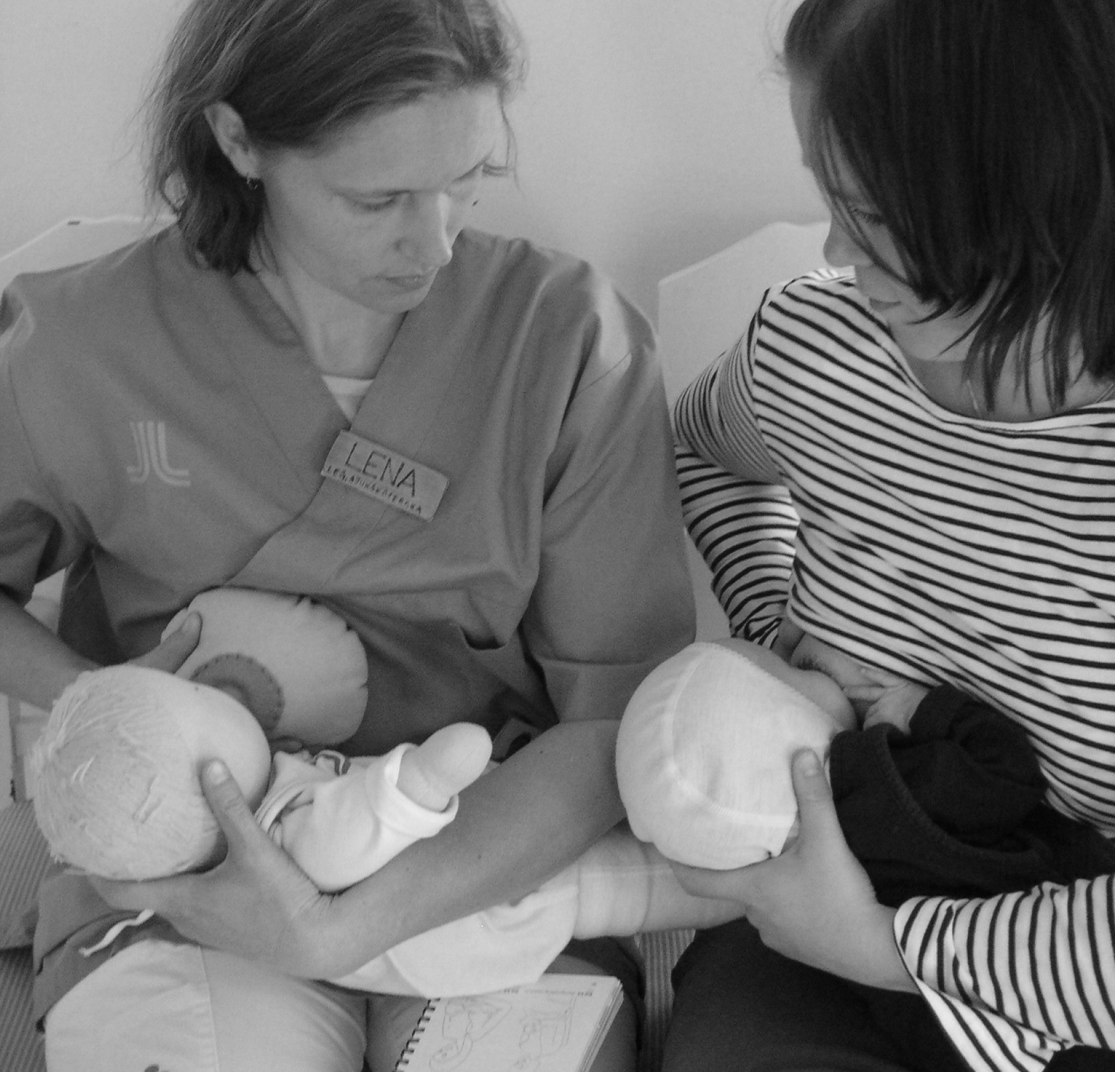
A teaching situation: Nurse with doll and artificial breast and mother with baby.

After the mothers have shared their spontaneous thoughts about the hands-on approach with the researcher, they were asked if they could suggest some alternatives. All the mothers described how they would have preferred the breastfeeding guidance given to them, for example, that nurses sit down besides them and spend more time sharing information and practical advice. The mothers were then questioned about the use of a doll and an artificial breast in order to demonstrate positioning and latching-on (See Figure 2.). No mother had even considered this alternative. Nine of them were positive about this visual alternative. Two mothers explained that this could also be a way of confirming that they had understood correctly.

"I have never thought about it. It might be an alternative, and perhaps already from the start ..."

After sharing their thoughts with the investigator, several mothers realized they perhaps would have needed a different type of support from nurses.

"I now realize that it was not exactly the type of help I needed."

The original research report illustrates more examples of each category [18].

## Discussion

According to Orem, the aim of nursing care with persons capable to act on their own care or that of their dependents, is to inform the persons, and to support them in their self-care capacities [37,38]. According to this, mothers should get individualized and respectful support to learn how to take care of their babies in NICU. In this study, most mothers described how they received care they did not understand, through professional behaviors they did not like, and that made them upset. According to Gustafsson, healthcare workers intend to perform good care but do not necessarily behave in such a way that achieves this [36]. In fact, some behaviors described by the mothers in this study appeared unacceptable to them. However, nurses were not questioned as to their behaviors and the meaning they give to them; this could be an interesting exploratory study to do. We already know that the environment in a NICU may not be optimal for nurses with all the premature or sick babies, and anxious parents. Nurses need to be knowledgeable in specialized and more medical type of neonatal care, but also in normal newborn care such as breastfeeding. However, babies in the NICU rarely behave like term healthy newborns when it comes to breastfeeding. So nurses have to be more knowledgeable but also more patient, with the babies and with the parents. Gustafsson [36] and Orem [37,38] argue that the nurse's role involves being sensitive to the parents' anxiety and need of support, that the nurse needs to help parents understand the difficulties their baby is experiencing and the different ways they could use to compensate or adjust to them. Aarts [39] would encourage nurses to teach parents in a very personalized manner so they can realize that there are many pathways to successful breastfeeding and that they will gradually find them with their baby and feeling more and more competent about it. In this study, most mothers felt that the permitting dialogue was missing, that they had not been informed about the hands-on approach so they could agree to it.

### Insult to integrity

The mothers reported about their experience from hands-on assistance in the breastfeeding situation, describing it as unpleasant, shocking, irritating, and disrespectful. We have not found any other study that reports mothers' experience with the hands-on approach or which has evaluated the mothers' feelings in that situation. Most were concerned with breastfeeding outcomes and not mothers' feelings. One of our basic questions remains unanswered: Is it possible that mothers' feelings in this type of situation explain the shorter period of breastfeeding when the staff use hands-on? We believe this practice is intrusive and disempowering for mothers, and that it could hinder their self-confidence to breastfeed, as Ingram and colleagues point out in the background of their article [13].

Orem suggests different care systems, of which the support and information system for persons capable to care for themselves and their dependents [37,38]. To enhance the mother's self-confidence, this type of care system is warranted. Nurses may spontaneously choose Orem's first system: the "doing it for the person who can't do it", by lack of time, as support and information are expected to take long time. However, they have to realize that the hands-on approach may be quicker to perform, but may hinder mother's confidence in her capacities in the long run. In fact, if more time was spent on support and information in the first place, mothers would feel respected and guided into becoming more competent to care for their special baby; they could quickly become more knowledgeable and more autonomous. In this study, it was the opposite for eight mothers out of ten; they felt their integrity was not respected by the hands-on approach which had not been explained before being performed.

### Understanding and adjustment

The eight mothers who reported that they did not agree with this spontaneous hands-on approach showed understanding and acceptance of the nurses' actions. This understanding could be explained by the mothers' dependence on nursing staff for this very special care their baby needed in the NICU, but it could also be explained by their wish for the best care possible for their fragile baby. According to Stern [2], in showing acceptance, mothers may try to protect this new life and in doing so, choose to trust the nurses' competence.

The mothers also reported that the hands-on assistance was often performed without information or teaching beforehand on how to help baby adequately to the breast, and even without previous attempts from their part to have the infant attached to the breast. They accepted the hands-on assistance as they did not know any alternative to this practice to help them with the new breastfeeding situation. The mothers in this study stressed that they needed the nurses' support and because of this, they chose not to say they did not appreciate the hands-on approach. They felt dependent so they adjusted to the nurses' behaviors. Armgard argues that by giving freedom to act, you participate in the exercise of power [43]. By not saying anything about what they disliked, the mothers did not have the opportunity to be involved in the decisions concerning their body and their wishes for positive experience in breastfeeding their fragile infant. We don't know of any study which reported similar feelings and experiences of mothers.

### Breasts as objects

Women and society in general, have given different meanings to breasts, and to breast touching by other people. Breasts are associated with the passage from girlhood to adulthood, sexual pleasure, and eroticism. But breasts also are inevitably linked to breastfeeding. Modern women often express feelings about their breasts [44], how they see them: too large, too small or not "good enough", and also how feminine or not feminine they feel when looking at them. From all times, breasts have been linked to both good and evil, leading to this ambivalent view of breasts as per their maternal and their sexual aspects [44]. Breasts having different meanings for different women, more attention need to be paid to professional behavior when teaching breastfeeding and especially latching-on to the breast.

Breast touching by other people is also very much a social act, generally accepted between intimate partners. The body itself, and not only breasts, is known to have its own territory (near zone) which cannot be crossed without permission, again often only by intimate partners. According to Norberg and colleagues, if this is not respected, the person will feel invaded and often react strongly or escape inside her body so not to be concerned about what happens on the body surface [45].

In this study, the hands-on assistance contributed to the mothers having an altered opinion of their own breasts. Mothers reported that their breasts became objects, handled by strangers in order to get baby to latch-on. They also felt their body territory was invaded but could not react too much to it since breasts now had become utilitarian in order to feed their newborn.

### Alternatives to hands-on support

All mothers expressed a need for assistance when starting breastfeeding, but could not suggest any alternative other than verbal explanations and more time devoted to be present near the mother. Maybe the fact that they understood and adjusted to this approach explains that they did not look for other ways to this teaching situation. When presented with the possibility of doing this type of teaching with a doll and a breast made of fabric, nine mothers agreed they would have preferred this method but had not thought about it. There are different ways to communicate, educate and inform mothers how they could support the babies to latch-on without the staff using hands-on. For example, nurses could use verbal information, videos about latch, group teaching, and so on [15]. The use of a doll and an artificial breast is easily acceptable within the framework used for this study. In fact, this method connects to Orem's information and support system since the purpose is to create a milieu that, through education, stimulates development [37,38]. This might lead to increasing confidence in the mother's own capacities and so increasing duration of breastfeeding and more satisfying relationship with the child during the breastfeeding experience. However, different options need to be explored more explicitly.

Some studies have reported verbal information and guidance with a breast made of fabric or seeing other mothers breastfeed (vicarious experience in the Bandura's model) decrease breastfeeding problems [11,16,17] and so possibly contribute to an increased duration of breastfeeding [10,13,14]. But more research needs to be done on educational methods suitable to teach latching-on especially with preterm or sick babies.

## Conclusion

This study increases information and insight about Swedish mothers' experiences with hands-on approach when breastfeeding a fragile infant in a NICU situation. Since most mothers in this study regarded this behavior as unpleasant and not helpful mostly because it was unexpected and unexplained, it would be important to either explain to mothers beforehand what type of physical approach could be attempted on their body, or better, to avoid this type of approach completely. This study also demonstrated that Swedish mothers react the same way to hands-on approach as mothers from cultures less supportive of breastfeeding. We suggest that nurses reflect on their behaviors while assisting mothers and think of alternatives to the hands-on help, such as the use of a doll and breast made of fabric. It is also important to educate the nurses in how to approach the mothers in a professional way [6,12]. Since the mothers were also shocked with the unexpected, it is mandatory that nurses take time to explain and support mothers in their breastfeeding experiences, so as to help them develop their self-care capacities and self-confidence. More research is needed pertaining to approaches for supporting and teaching parents in breastfeeding situations with fragile infants in NICU.

This study also emphasizes the importance of basing nursing research on nursing theoretical frameworks in order to guide reflection and action. It has contributed to increased knowledge in three nursing theories, Gustafsson's, Orem's, and Aart's, and about interrelations between those three theories. We have shown that they complete each other in explaining the capacities of the person, the nurse's role in supporting mothers' confidence and autonomy, and professional approaches to attain this goal. More research needs to be done in this area.

## Abbreviations

NICU – Neonatal Intensive Care Unit

WHO – World Health Organization

UNICEF – The United Nations Children's Fund

SAUC – Sympathy Acceptance Understanding Competence

## Competing interests

The author(s) declare that they have no competing interests.

## Authors' contributions

LW designed and conducted study as a partial requirement for a master's degree; she was then assisted by her director, VW, and her co-director, KS. LD has critically analyzed the interrelations between the three conceptual models, and reviewed the description of the qualitative method and the interpretation of the results from the interviews. LW, KS, LN and LD wrote the manuscript for publication. LN has worked on the editing of the manuscript and in obtaining background information from the NICU. All authors read and approved the final manuscript.

## References

[B1] Gauthier I, Dumas L (2002). La méthode kangourou pour faciliter l'attachement. (The kangaroo method to facilitate attachment) L'Infirmière Canadienne.

[B2] Stern D (1998). The Birth of a Mother.

[B3] Lawrence RA (2005). Breastfeeding – A Guide for the Medical Profession.

[B4] Riordan J, Auerbach KG (2005). Breastfeeding and Human Lactation.

[B5] Khoso M (2000). Ingen amma-mamma – kvinnors upplevelser av amningssvårigheter som lett till amningsavbrytande (No breastfeeding mother – women's experiences of difficulties that leads to interruption of breastfeeding). Institutet för psykologi (Institute of psychology) Lunds Universitet, Sweden.

[B6] Ekström A, Widström AM, Nissen E (2005). Process oriented training in breastfeeding alters attitudes to breastfeeding in health professionals. Scand J Public Health.

[B7] Porteous R, Kaufman K, Rush J (2000). The effect of individualized professional support on duration of breastfeeding: A randomized controlled trial. J Hum Lact.

[B8] Blyth R, Creedy D, Dennis CL, Moyle W, Pratt J, De Vries SM (2002). Effect of maternal confidence on breastfeeding duration: an application of breastfeeding self-efficacy theory. Birth.

[B9] Pantazi M, Jaeger MC, Lawson M (1998). Staff support of mothers to provide breastmilk in pediatric hospital and neonatal units. J Hum Lact.

[B10] Cox SG, Turnbull CJ (1998). Developing effective interactions to improve breastfeeding outcome. Breastfeed Rev.

[B11] Duffy EP, Percival P, Kershaw E (1997). Positive effects of an antenatal group teaching session on postnatal nipple pain, nipple trauma and breastfeeding. Midwifery.

[B12] Hedberg-Nyqvist K, Sjödén PO, Ewald U (1994). Mothers' advice about facilitating breastfeeding in neonatal intensive care unit. J Hum Lact.

[B13] Ingram J, Johnson D, Greenwood R (2002). Breastfeeding in Bristol: teaching good positioning, and support from fathers and families. Midwifery.

[B14] Fletcher D, Harris H (2000). The implementation of the HOT program at the Royal Women's Hospital. Breastfeed Rev.

[B15] Fairbank L, O'Meara S, Renfrew MJ, Woolridge M, Sowden AJ, Lister-Sharp D (2000). A systematic review to evaluate the effectiveness of interventions to promote the initiation of breastfeeding. Health Technol Assess.

[B16] Bassett V, Dumas L, Mayrand Leclerc M (2002). A prenatal intervention focused on influencing postpartum breastfeeding confidence. Ninth International Conference of Maternity Care Researchers; April 2002; North Carolina.

[B17] Dennis CL (1999). Theoretical underpinnings of breastfeeding confidence: a self-efficacy framework. J Hum Lact.

[B18] Weimers L, Wahlberg V, Svensson K (2002). Upplevelser av information och handgriplig hjälp i amningssituationen En kvalitativ studie med djupintervjuer av 12 mödrars vars barn vårdats på en neonatalavdelning. (Experiences of information and physical help in breastfeeding – A qualitative study consistingof in-depth interviews of 12 mothers whose babies have been on a neonatal ward). Master thesis Ersta Sköndal Högskola, Sweden, Institution för vårdvetenskap.

[B19] World Health Organization/UNICEF (1989). Protecting, Promoting and Support Breastfeeding: The Special Role of Maternity Services A Joint WHO/UNICEF Statement.

[B20] World Health Organization/UNICEF (1990). The Innocenti Declaration on the Protection, Promotion, and Support of Breastfeeding.

[B21] World Health Organization (1981). WHO International Code of Marketing of Breast-milk Substitutes.

[B22] Wheeler J, Chapman C, Johnson M, Langdon R (2000). Feeding outcomes and influences within the Neonatal Unit. Int J Nurs Pract.

[B23] Winberg J (2005). Mother and newborn baby: Mutual regulation of physiology and behavior-A selective review. Dev Psychobiol.

[B24] Carfoot S, Williamson P, Dickson R (2005). A randomised controlled trial in the north of England examining the effects of skin-to-skin care on breastfeeding. Midwifery.

[B25] Bystrova K, Widström AM, Matthiesen AS, Ransjö-Ardvidson AB, Welles-Nyström B, Wassberg C, Vorontsof I, Uvnäs-Moberg K (2003). Skin-to-skin contact may reduce the negative consequences of "the stress of being born": a study on temperature in newborn infants, subjected to different ward routines in St.Petersburg. Acta Paediatr.

[B26] Matthiesen A-S, Ransjö-Arvidson A-B, Nissen E, Uvnäs-Moberg K (2001). Postpartum maternal oxytocin release by newborns: Effect of infant hand massage and sucking. Birth.

[B27] Charpak N, Ruiz JG, Zupan J, Cattaneo A, Figueroa Z, Tessier R, Cristo M, Anderson G, Ludington S, Mendoza S, Mokhachane M, Worku B (2005). Kangaroo mother care: 25 years after. Acta Paediatr.

[B28] Mikiel-Kostyra K, Mazur J, Boltruszko I (2002). Effect of early skin-to-skin contact after delivery on duration of breastfeeding: a prospective cohort study. Acta Pediatrica.

[B29] Lang S (1997). Breastfeeding Special Care Babies.

[B30] Dodd VL (2005). Implications of kangaroo care for growth and development in preterm infants. J Obstet Gynecol Neonatal Nurs.

[B31] Weimers L, Nyström H (2000). Amningsguiden En Guiden om Amning för Sjuka och Underburna Barn, utifrån WHO/UNICEF:s 10 Steg till en Lyckad Amning (Breastfeeding Guide – A Guide about Breastfeeding Sick and Preterm Children Based on WHO/UNICEF:s 10 Steps to Successful Breastfeeding).

[B32] Vannuchi MT, Monteiro CA, Rea MF, Andrade SM, Matsuo (2004). The Baby-Friendly Hospital Initiative increases breastfeeding in neonatal unit. Rev Saude Publica.

[B33] Bicalho-Mancini PG, Velasquez-Melendez G (2004). Exclusive breastfeeding at the point of discharge of high-risk newborns at a neonatal intensive care unit and the factors associated with this practice. J Pediatr (Rio J).

[B34] Merewood A, Philipp BL, Chawal N, Cimo S (2003). The Baby-Friendly Hospital Initiative increases breastfeeding rates in US neonatal intensive care unit. J Hum Lact.

[B35] Gustafsson B (2003). Nurses' self-relation – Becoming theoretically competent: The SAUC model for confirming nursing. Nurs Sci Q.

[B36] Gustafsson B (1997). Bekräftande Omvårdnad -SAUK-modellen för Vård och Omsorg (Confirming Nursing The SAUC Model for Health and Community Care).

[B37] Orem ED (1980). Nursing: Concepts of Practice.

[B38] Dumas L (1990). Choisir un cadre théorique. Choisir Orem (Choosing a theoretical framework. Choosing Orem). Nurs Que.

[B39] Aarts M (2000). Aarts M Marte Meo: Basic Manual.

[B40] Radnitzky G (1970). Contemporary Schools of Metascience.

[B41] Lincoln YS, Guba E (1985). Naturalistic Inquiry.

[B42] Tobin GA, Begley CM (2004). Methodological rigour within a qualitative framework. J Adv Nurs.

[B43] Armgard L-O (1993). Antropologi Problem i KE Lögstrups Författarskap (Anthropology Problems in KE Lögstrups Writings).

[B44] Yalom M (1997). A History of the Breast.

[B45] Norberg A, Axelsson K, Rahm Hallberg I, Lundman B, Athlin E, Ekman S-L, Engström B, Jansson L, Kihlgren M (1992). Omvårdnadens Mosaik(The Mosaic of Care).

